# *Cryptosporidium* spp. in wild murids (Rodentia) from Corsica, France

**DOI:** 10.1007/s00436-021-07369-4

**Published:** 2021-11-24

**Authors:** Katherine García-Livia, Ángela Fernández-Álvarez, Carlos Feliu, Jordi Miquel, Yann Quilichini, Pilar Foronda

**Affiliations:** 1 Instituto Universitario de Enfermedades Tropicales Y Salud Pública de Canarias, Universidad de La Laguna, San Cristóbal de La Laguna, Tenerife, Canary Islands Spain; 2 Department Obstetricia Y Ginecología, Pediatría, Medicina Preventiva Y Salud Pública, ToxicologíaMedicina Legal Y Forense Y Parasitología, Universidad de La Laguna, San Cristóbal de La Laguna, Tenerife, Canary Islands Spain; 3 UMR SPE 6134, CNRS-Université de Corse, Projet GEM, Campus Grimaldi Bât 018, 20250 Corte, France; 4 Department Biologia, Sanitat i Medi Ambient, Facultat de Farmàcia I Ciències de L’Alimentació, Universitat de Barcelona, Barcelona, Spain; 5 Institut de Recerca de La Biodiversitat (IRBio), Facultat de Biologia, Universitat de Barcelona, Barcelona, Spain

**Keywords:** *Rattus rattus*, *Rattus norvegicus*, *Mus musculus domesticus*, *Apodemus sylvaticus*, *Cryptosporidium viatorum*, Corsica

## Abstract

*Cryptosporidium* spp. are worldwide protozoan parasites that can affect to a broad range of vertebrate hosts, including rodents. In the island of Corsica (France), there are no previous data about these protozoa infecting wild rodents. To estimate the distribution and occurrence, a total of 117 wild murine rodents of the species *Rattus rattus* (84), *Mus musculus domesticus* (21), *Apodemus sylvaticus* (11), and *Rattus norvegicus* (1) were captured in 24 different biotopes. Fecal samples were screened for *Cryptosporidium* spp. by nested PCR to amplify an 830 bp fragment of the 18S rRNA gene. As general occurrence, 15.4% of the rodents analyzed were positive for *Cryptosporidium* spp., being detected widely distributed along the island in *R. rattus* (17.6%) and *M. m. domesticus* (14.3%). *Cryptosporidium viatorum*, *Cryptosporidium* sp. rat genotype II, and *Cryptosporidium* sp. rat genotype III were successfully identified in *R. rattus*. The results herein reported provide the first data on *Cryptosporidium* spp. in wild murine species from a Mediterranean island and constitute the first report of the zoonotic species *C. viatorum* in *R. rattus*. Although a low occurrence of *Cryptosporidium* spp. in murids was obtained and only in one animal the zoonotic species *C. viatorum* was identified, our results highlight that wild murine rodents from Corsica could mediate in the maintenance and transmission of this protozoan to the environment and other hosts including humans and animals. Further studies are required to better understand the epidemiology of *Cryptosporidium* spp. in wild rodents from Corsica and their possible public health repercussions.

## Introduction

The protozoan *Cryptosporidium* spp. (phylum Apicomplexa) infects a broad range of vertebrate hosts that can play an important role in the maintenance and transmission of this pathogen (Kváč et al. 2014). It can be found in more than 150 mammalian species (Bauerfeind et al. 2016), being the order Rodentia, which represents at least 43% of the mammalian species (Wilson and Reeder 1993; Huchon et al. 2002), the most abundant and diversified order of mammals considered to be reservoirs of *Cryptosporidium* spp. (Feng 2010). The morphological and biological adaptations of rodents allow them to survive in any type of environments (Huchon et al. 2002), being significantly more abundant in anthropogenically modified habitats (e.g., agricultural lands, pasturelands, urban areas) than non-modified habitats (Mendoza et al. 2020). This ability facilitates rodents to spread and transfer their pathogens, such as *Cryptosporidium* spp., to humans and wild and domestic animals in rural and urban areas (Meerburg et al. 2009). *Cryptosporidium* spp. are the causal agent of cryptosporidiosis, a leading cause of diarrheal disease in both humans and animals worldwide (Innes et al. 2020). This protozoan can be transmitted by fecal–oral contamination, via ingestion of contaminated water or food, direct contact with infected persons or animals, indirect contact with contaminated fomites, and, in some cases, via inhalation (Sponseller et al. 2014; Bauerfeind et al. 2016).

The prevalence of *Cryptosporidium* spp. infection in rodents is highly variable worldwide (Feng 2010). A recent systematic review and meta-analysis estimated a 17% of pooled global prevalence of *Cryptosporidium* spp. infection in rodents, obtaining a 22% of prevalence in Europe (see Taghipour et al. 2020). In fact, previous studies reported prevalence rates of *Cryptosporidium* spp. infection in rodents ranging between 8.0 and 31.4% in mice and 2.1 and 63.0% in rats (Feng 2010; Koehler et al. 2018; Zhao et al. 2018, 2019). Currently, more than 40 *Cryptosporidium* species and similar number of genotypes have been recognized as valid worldwide (Zahedi and Ryan 2020; Ježková et al. 2020; Zahedi et al. 2021). At least 21 *Cryptosporidium* species and 21 genotypes have been cited in rodents: *Cryptosporidium muris*, *Cryptosporidium parvum*, *Cryptosporidium hominis*, *Cryptosporidium meleagridis*, *Cryptosporidium tyzzeri*, *Cryptosporidium ubiquitum*, *Cryptosporidium suis*, *Cryptosporidium scrofarum*, *Cryptosporidium erinacei*, *Cryptosporidium canis*, *Cryptosporidium wrairi*, *Cryptosporidium rubeyi*, *Cryptosporidium andersoni*, *Cryptosporidium proliferans*, *Cryptosporidium occultus*, *Cryptosporidium viatorum*, *Cryptosporidium ditrichi*, *Cryptosporidium apodemi*, *Cryptosporidium alticolis*, *Cryptosporidium microti*, *Cryptosporidium ratti*, rat genotypes II–IV, mouse genotypes II and III, naruko genotype, ferret genotype, chipmunk genotypes I and II, skunk genotype, hamster genotype, deer mouse genotypes I–IV, vole genotype, bear genotype, muskrat genotypes I and II, and ground squirrel genotypes I–III (see Lv et al. 2009; Paparini et al. 2012; Ng-Hublin et al. 2013; Kváč et al. 2014; Čondlová et al. 2018; Koehler et al. 2018; Zhang et al. 2018; Zhao et al. 2018; Horčičková et al. 2019; Zahedi and Ryan 2020; Ježková et al. 2020). Among them, *C. parvum* and *C. hominis* are the major species involved in human and waterborne outbreaks (Xiao 2010; Ryan and Xiao 2014). *Cryptosporidium parvum*, *C. muris*, and *Cryptosporidium* sp. rat genotype III are the species frequently detected in rats (Koehler et al. 2018), being *C. muris* and *Cryptosporidium* sp. mouse genotype I the most reported species in mice (Morgan et al. 1999, 2000; Foo et al. 2007).

**Fig. 1:**
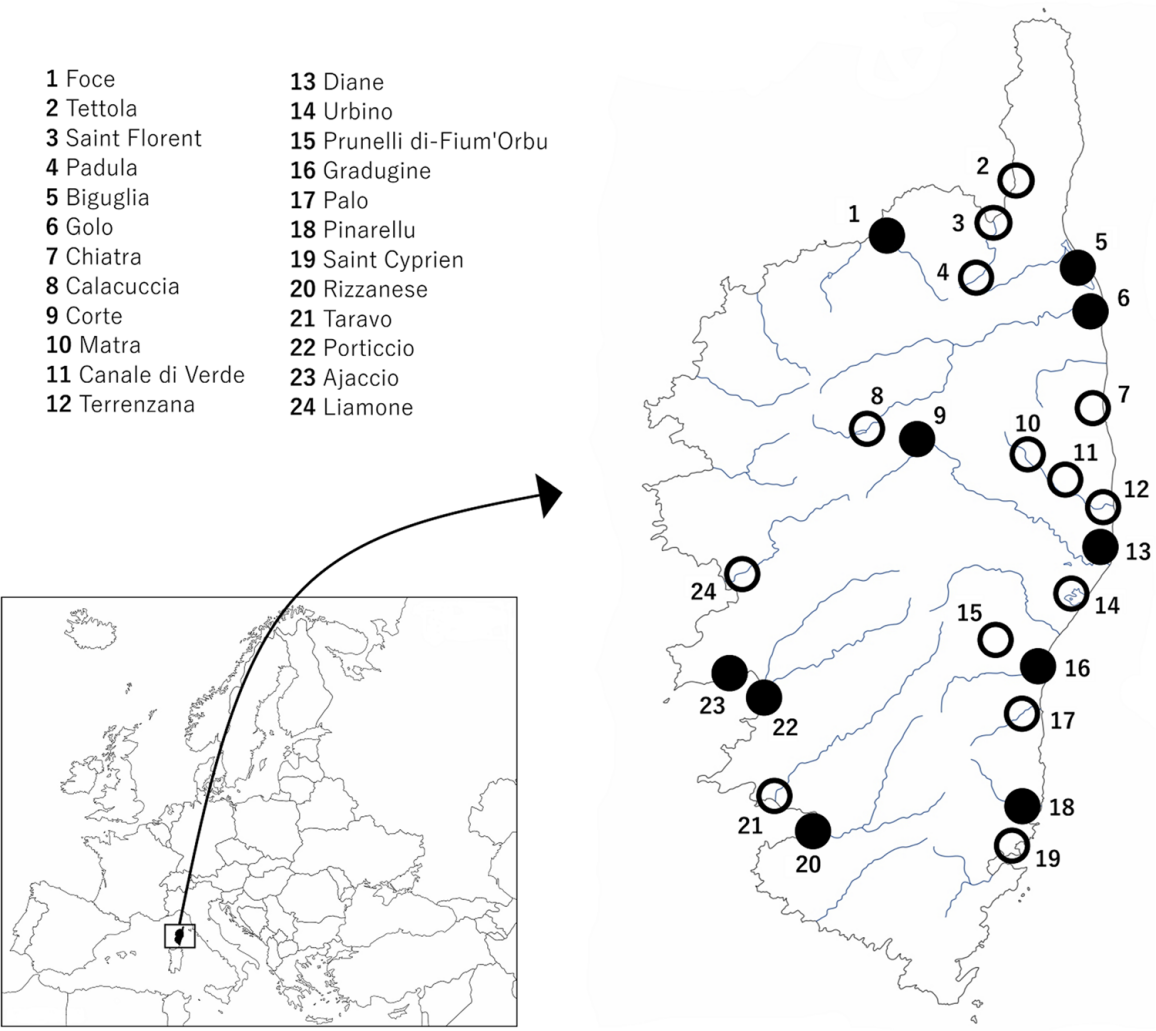
Geographical location of Corsica (France) and map of the island showing the distribution of rodent sampling locations (numbers 1–24). The presence of *Cryptosporidium* spp. in wild rodents are represented in a black circle. (The original images were taken from d-maps (https://d-maps.com/carte.php?num_car=2801&lang=es, https://d-maps.com/carte.php?num_car=2233&lang=es) in which the original author authorized its free use for any purpose. Both images were edited by Paint 3D program)

Corsica (France), considered the fourth largest Mediterranean island and the most mountainous and forested one (Grech-Angelini et al. 2016), is situated in the southeast of the French mainland and west of the Italian Peninsula (Fig. 1). This well-known tourist destination is characterized by a mild Mediterranean climate and a high variability of microclimates (Grech-Angelini et al. 2016), which allows the development of a large diversity of natural environments where many animal species, including rodents, are well established. In terms of public health and veterinary, available data about the pathogens harboring wild murine rodents from Corsica are based on *Fasciola hepatica* in *Rattus rattus* and *Mus musculus* (Valero et al. 1998, 2002; Ménard et al. 2000; Magnanou et al. 2006), *Schistosoma* spp. in *R. rattus* (Oleaga et al. 2019), and *Borrelia burgdorferi* sl in *Ixodes ricinus* ticks of *R. rattus* (Cicculli et al. 2019). Within a multidisciplinary study, the species *Coxiella burnetii* and *Toxoplasma gondii* in *R. rattus* and *M. m. domesticus* (Izquierdo-Rodríguez et al. 2019) and pathogenic *Leptospira* species in *R. rattus*, *Mus musculus domesticus*, *Rattus norvegicus*, and *Apodemus sylvaticus* (Izquierdo-Rodríguez et al. 2020) were detected. As a part of this multidisciplinary study, and considering the lack of data on *Cryptosporidium* spp. in rodents from Corsica, we aimed to analyze its distribution, occurrence, and identity of the species and genotypes present in wild murids from Corsica.

## Materials and methods

### Sample collection

From a multidisciplinary study carried out in Corsica (France) between February and June 2016, a total of 117 wild murine rodents of the species *R. rattus* (black rat) (*n* = 84), *M. m. domesticus* (domestic mouse) (*n* = 21), *A. sylvaticus* (wood mouse) (*n* = 11), and *R. norvegicus* (gray rat) (*n* = 1) were captured alive using live traps. With the aim of collecting animals from different biotopes, traps were set in 24 municipalities along Corsica considering North and South of the island following Izquierdo-Rodríguez and collaborators (Izquierdo-Rodríguez et al. 2020) (Table 1), mainly at suburban-rural sites near ponds, river mouths, and lakes (Fig. 1). Euthanasia was performed with CO_2_ inhalation or by cervical dislocation, and, after dissection, a portion of fecal samples from the rectum of each animal were preserved in 2.5% potassium dichromate and conserved until examination. This study was carried out in strict accordance with the recommendations of the guidelines of animal welfare in experimental science and the European Union legislation (Directive 86/609/EEC). The protocol was approved by the Ethics Committee of Investigation and Animal Wellness of Universidad de La Laguna (register number CEIBA2018-0330).

**Table 1:** Rodent species analyzed for the detection of *Cryptosporidium* spp., as well as the locations where they have been captured in Corsica. All species/genotypes identified were detected in *R. rattus*. Abbreviations: *%* occurrence of *Cryptosporidium* spp., + */n* positive animals for *Cryptosporidium* spp./number of samples analyzed (* one *Rattus norvegicus*)

Locations	*Rattus rattus* (+ /*n*)	*Mus musculus domesticus* (+ /*n*)	*Apodemus sylvaticus* (+ /*n*)	Total (+ /*n*)	Species/genotype
North					
Biguglia	2/3	0/2	0/0	2/5	*Cryptosporidium* sp. rat genotype II (*n* = 1)
Calacuccia	0/3	0/1	0/2	0/6	
Canale di Verde	0/5	0/2	0/0	0/7	
Chiatra	0/1	0/0	0/0	0/1	
Corte	2/5	0/3	0/3	2/11	*Cryptosporidium* sp. rat genotype II (*n* = 1)
Diane	2/5	0/1	0/0	2/6	*Cryptosporidium* sp. rat genotype II (*n* = 2)
Foce	2/7	0/2	0/1	2/10	
Golo	0/3*	1/3	0/0	1/6	
Gradugine	1/3	0/0	0/0	1/3	
Matra	0/1	0/0	0/1	0/2	
Padula	0/2	0/0	0/0	0/2	
Palo	0/3	0/1	0/0	0/4	
Prunelli di-Fium’Orbu	0/5	0/0	0/0	0/5	
Saint Florent	0/5	0/0	0/2	0/7	
Terrenzana	0/6	0/1	0/0	0/7	
Tettola	0/0	0/0	0/2	0/2	
Urbino	0/5	0/0	0/0	0/5	
Total (+ /*n*) (%)	9/62 (14.5)	1/16 (6.2)	0/11 (0)	10/89 (11.2)	
South					
Ajaccio	1/4	0/0	0/0	1/4	
Liamone	0/6	0/1	0/0	0/7	
Pinarellu	1/1	2/2	0/0	3/3	*Cryptosporidium* sp. rat genotype II (*n* = 1)
Porticcio	2/2	0/0	0/0	2/2	*Cryptosporidium* sp. rat genotype II (*n* = 1)
Rizzanese	2/5	0/0	0/0	2/5	*Cryptosporidium viatorum* (*n* = 1) *Cryptosporidium* sp. rat genotype III (*n* = 1)
Saint Cyprien	0/2	0/1	0/0	0/3	
Taravo	0/3	0/1	0/0	0/4	
Total (+ /*n*) (%)	6/23 (26)	2/5 (40)	-	8/28 (28.6)	
Total (+ /*n*) (%)	**15/85 (17.6)**	**3/21 (14.3)**	**0/11 (0)**	**18/117 (15.4)**	

### DNA isolation

Before DNA extraction, 200 µl of the samples was washed with PBS-EDTA at room temperature to remove the potassium dichromate. Next, total DNA was isolated directly by following the manufacturer’s instructions of the commercial FastDNA SPIN kit (Qbiogene, Illkirch Cedex, France) using the TissueLyser II (Qiagen, Hilden, Germany) as oocyst disruptor. DNA was stored at − 20 °C until further processing.

### PCR amplification

*Cryptosporidium* spp. were detected by nested PCR targeting an 830 bp of the 18S ribosomal rRNA gene using the primer pair SSU-F1/SSU-R1 and SSU-F2/SSU-R2 for the primary and secondary PCR, respectively (Zhao et al. 2013). The set-up of the PCR reactions was carried out according to the method of Zhang and collaborators (Zhang et al. 2015). The reaction mixture for all pairs of primers contained 0.625 U Taq DNA polymerase, 0.4 µM of each primer, 200 µM each dNTPs, 2 mM MgCl2, 1 × buffer (Mg2 + free), 2 µl of DNA template, and water to a total volume of 25 µl. The cycling conditions for both amplifications were initial denaturation of 95 °C for 5 min followed by 35 cycles 94 °C for 45 s, 45 s at suitable temperature (55 °C for primary PCR and 58 °C for secondary PCR), and 1 min at 72 °C, followed by a final extension step at 72 °C for 10 min. All PCR reactions were performed in a Labnet Thermocycler (Labnet International, Berkshire, UK). PCR products were resolved on 1.5% agarose gels, and PCR-positive products were purified using the UltraClean PCR Clean-up kit (Mo-Bio Laboratories, Inc., Carlsbad, CA, USA). In cases where bands of different sizes occurred, the desired size band was cut and purified with the QIAEX® II Gel Extraction kit (Qiagen, Hilden, Germany). In both cases, the manufacturer’s recommendations were followed.

### Sequencing and phylogenetic analyses

The purified PCR-positive products were sequenced at Macrogen Europe (Amsterdam, the Netherlands). Nucleotide sequences obtained were edited with the MEGA X program (Kumar et al. 2018) and subsequently aligned with the ClustalW program included in MEGA X. Minor corrections, to increase the aligned sequence similarity and improve the inferences on any positional homology, were then made by hand. A BLAST search was carried out in order to elucidate any homologies or similarities with the sequences previously published in the GenBank database. The molecular identification was achieved by phylogenetic analysis through the neighbor-joining distance method (Saitou and Nei 1987) with at least 1000 bootstrap replications. Nucleotide sequences obtained in this work and reference sequences from other *Cryptosporidium* species and genotypes from the GenBank were aligned. *Toxoplasma gondii* was used as the outgroup. The obtained sequences generated in this study have been deposited in GenBank under the accession numbers MW590662–MW590669.

### Statistical analyses

A chi-square test, setting the *P* value in 0.05, was conducted for the comparison of *Cryptosporidium* spp. prevalence obtained between the rodent species and the capture area, North and South. Data analysis was carried out using SPSS v22.0 statistical software.

## Results

In this study, *Cryptosporidium* DNA was detected in wild murids fecal samples, and it was found widely distributed along Corsica, being detected both in the North and the South of the island homogeneously with 11.2% and 28.6% of occurrence, respectively, without statistical differences (Fig. 1, Table 1). Of the 117 wild murine rodents analyzed, 18 resulted positive for the amplification of the18S rRNA gene fragment with a general occurrence of 15.4% (Table 1). Considering the four murine species analyzed, *R. rattus* and *M. m. domesticus* were the only species found infected by *Cryptosporidium* spp. Of the 18 PCR-positive samples, 15 correspond to rats, nine of them were captured in the North of Corsica, specifically in the regions of Diane (*n* = 2), Gradugine (*n* = 1), Foce (*n* = 2), Corte (*n* = 2), and Biguglia (*n* = 2). The other six rats were captured in the South regions of Porticcio (*n* = 2), Ajaccio (*n* = 1), Pinarellu (*n* = 1), and Rizzanese (*n* = 2). In the case of mice, three resulted infected by *Cryptosporidium* spp., two of them were captured in the South region of Pinarellu and the other one was captured in the North region of Golo (Fig. 1). No significant differences were found in the occurrence of murine species infected between North and South of Corsica. The general occurrences of infection in *R. rattus* and *M. m. domesticus* were 17.6% (15/85) and 14.3% (3/21), respectively (Table 1).

**Fig. 2:**
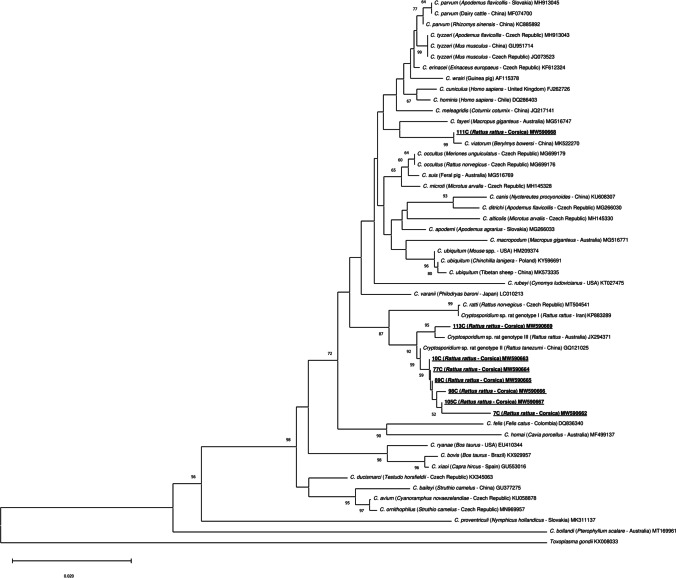
Phylogenetic analysis using the neighbor-joining method with p-distance and 1000 bootstrap replications based on the 18S ribosomal RNA fragment (830 bp). *Toxoplasma gondii* was used as the outgroup

In this study, only eight PCR-positive samples from *R. rattus* were successfully sequenced (Table 1). Phylogenetic analysis showed the presence of 3 *Cryptosporidium* species and genotypes (Fig. 2), including *Cryptosporidium* sp. rat genotype II, *Cryptosporidium* sp. rat genotype III, and the zoonotic species *C. viatorum*. *Cryptosporidium* sp. rat genotype II was the most detected genotype, which has been found widely distributed in North and South of Corsica, while *Cryptosporidium* sp. rat genotype III was only detected in one rat in the South, as was *C. viatorum*. A sequence obtained from *R. rattus*, captured in the South region of Rizzanese (isolate 111C), clustered together with a *C. viatorum* sequence obtained from *Berylmys bowersi* from China (GenBank: MK522270). Additionally, six sequences from *R. rattus*, captured in the North regions of Diane (isolates 7C and 10C), Corte (isolate 89C), and Biguglia (isolate 105C), and in the South regions of Ajaccio (isolate 77C) and Pinarellu (isolate 98C), clustered together with *Cryptosporidium* sp. rat genotype II from *Rattus tanezumi* from China (GenBank: GQ121025). One sequence from *R. rattus*, captured in the South region of Rizzanese (isolate 113C), clustered together with *Cryptosporidium* sp. rat genotype III from *R. rattus* from Australia (GenBank: JX294371).

Four different haplotypes of *Cryptosporidium* sp. rat genotype II were detected. The most prevalent, haplotype 1, was found in the North (isolates 10C and 89C) and South (isolate 77C) of Corsica. One second haplotype, haplotype 2, was identified in the North of the island (isolate 105C), whose nucleotide sequence presented two extra adenine bases in positions 172 and 173 with respect to haplotype 1. In the case of haplotype 3, detected in Southern Corsica (isolate 98C), it presented a base pair change of T→A and C→A at position 63 and 235 with respect to haplotype 1, respectively. In the haplotype 4, detected in Northern Corsica (isolate 7C), the nucleotide sequenced presented a base pair change of T→G in position 160, A→C in position 161, and C→G in positions 158 and 246.

## Discussion

This is the first epidemiology survey of *Cryptosporidium* spp. in murine rodents from Corsica. A total of three *Cryptosporidium* species and genotypes, concretely, *Cryptosporidium* sp. rat genotype II, *Cryptosporidium* sp. rat genotype III, and *C. viatorum*, were successfully identified with a 15.4% of occurrence. The occurrences obtained for rats (17.6%) and mice (14.3%) are within the reported ranges for mice (8.0–31.4%) and rats (2.1–63.0%) (Feng 2010; Koehler et al. 2018; Zhao et al. 2018, 2019).

The most prevalent genotype was *Cryptosporidium* sp. rat genotype II, contrary to the genotype found in other studies where *Cryptosporidium* sp. rat genotype III is the most frequently reported (Koehler et al. 2018). *Cryptosporidium* sp. rat genotype II was found in six rats widely distributed both in the North and South of the island of Corsica. However, *Cryptosporidium* sp. rat genotype III and *C. viatorum* were only found in the South of the island, but as they have been detected in a single animal each, this is not a congruent result to assess if there are any difference between their distribution on the island. Therefore, more studies are required to search for these species and genotypes in the North of the island, considering the few animals from which we were able to successfully sequence *Cryptosporidium* spp.

*Cryptosporidium* sp. rat genotype II and *Cryptosporidium* sp. rat genotype III are host-adapted genotypes (Zahedi et al. 2016) that have been detected in rats worldwide (Lv et al. 2009; Paparini et al. 2012; Ng-Hublin et al. 2013; Koehler et al. 2018; Tan et al. 2019; Zhao et al. 2019; García-Livia et al. 2020). Nevertheless, other hosts can be infected by *Cryptosporidium* sp. rat genotypes II and III, as has been reported in mice, cats, sheep, and goats (Ryan et al. 2005; Lv et al. 2009; Paparini et al. 2012; Ng-Hublin et al. 2013; Koinari et al. 2014; Yang et al. 2015; Hatam-Nahavandi et al. 2019). To date, the potential of the rat genotypes identified in this study to cause cryptosporidiosis in livestock or humans remains unclear.

*Cryptosporidium viatorum*, the only zoonotic species identified in this study, has been originally found exclusively in humans (Elwin et al. 2012; Insulander et al. 2013; Lebbad et al. 2013; Adamu et al. 2014; Ayinmode et al. 2014; Stensvold et al. 2015; De Lucio et al. 2016; Sánchez et al. 2017; Ukwah et al. 2017) and in urban wastewater and combined sewer overflows in China (Huang et al. 2017). Recently in 2018, *C. viatorum* was reported in a non-human host, concretely in the wild rats *Rattus lutreolus* from Australia (Koehler et al. 2018), and in the species *R. norvegicus*, *Leopoldamys edwardsi*, and *B. bowersi* from China (Zhao et al. 2019; Chen et al. 2019). Therefore, our study constitutes the first report of *C. viatorum* in *R. rattus* and the fifth report in non-human hosts, expanding its host range. Our results support recent findings suggesting that wild murines could be hosts of the zoonotic species *C. viatorum*. In fact, *C. viatorum* XVa subtype family from wild rats has been recently reported to be genetically identical to those subtypes found in humans (Chen et al. 2019). Further molecular investigations are needed to better understand the epidemiology of *C. viatorum* and clarify its transmission routes.

Considering that the species *C. viatorum* was the only zoonotic species detected in a single animal, more analyses are required to determine the occurrence of this and other possible zoonotic *Cryptosporidium* species that could have not been detected in wild murids from Corsica. In this sense, it is important to note that a recent study, conducted between 2017 and 2019 with 750 online reports from the National Reference Center-Expert of cryptosporidiosis in France, showed that the 40% of the reported cases of human cryptosporidiosis in this country were mainly related to recreational water (48%) and animal contact (23%) (Costa et al. 2020). In Corsica, no cases of cryptosporidiosis in humans have been reported to date, but considering that murine rodents can maintain the transmission cycles of several *Cryptosporidium* zoonotic species, such as *C. parvum*, *C. muris*, *C. ubiquitum*, *C. meleagridis*, *C. scrofarum*, *C. viatorum*, *C. canis*, *C. tyzzeri*, *C. andersoni*, *C. hominis*, *C. suis*, *C. proliferans*, *C. occultus*, *C. wrairi*, *C. rubeyi*, and *C. ditrichi* (Zhao et al. 2018; Koehler et al. 2018; Zhang et al. 2018; Beser et al. 2020), the detection of *C. viatorum* in this study should not be dismissed.

This study constitutes the first survey of *Cryptosporidium* spp. infection in wild murine species from a Mediterranean island. Some studies have previously confirmed the presence of this protozoan in murine rodent species from Atlantic islands (Feliu et al. 2012; García-Livia et al. 2020), showing that *Cryptosporidium* spp. infection is common in *R. rattus* and *M. m. domesticus* from the Canary Islands (Spain), with a high diversity of *Cryptosporidium* species and genotypes. In Tenerife (Canary Islands), a total of seven *Cryptosporidium* species/genotypes were identified, while in Corsica, despite being an island much larger in size than Tenerife and both studies with similar sample size (*n* = 97 in Tenerife and *n* = 1117 in Corsica), a low occurrence was obtained and a total of three *Cryptosporidium* species/genotypes were identified. Further studies analyzing more samples are required in order to obtain additional data about the occurrence and biodiversity of *Cryptosporidium* spp. in wild murids from Corsica.

Factors such as host specificity, ecological interactions, geographic distribution, bioclimatic conditions, and sampled areas, among others, could influence in the occurrence and prevalence of *Cryptosporidium* spp. in wild rodents (Zhao et al. 2019). Considering that samples of our study were only obtained near ponds, river mouths, and lakes, together with the fact that only few samples were successfully sequenced, further investigations with an increased in the number of samples and in non-analyzed areas would improve the knowledge about the prevalence, distribution, and biodiversity of *Cryptosporidium* spp. in wild murine rodents from Corsica.

## Conclusions

The present study constitutes the first report of *Cryptosporidium* spp. in wild murids from Corsica (France), thus creating an overview of the epidemiological situation of this parasite in this region. *Cryptosporidium* sp. rat genotype II, *Cryptosporidium* sp. rat genotype III, and *C. viatorum* were the *Cryptosporidium* species and genotypes successfully identified in fecal samples from wild murine rodents, being the first citation of *C. viatorum* in *R. rattus*.

Considering the interactions between wildlife, livestock, and humans, further investigations should be carried out in unsampled areas, in addition to other wild hosts, to better understand the epidemiology of *Cryptosporidium* spp. in Corsica and to determine the possible zoonotic risks of transmission.

## Data Availability

The dataset used and analyzed by the authors during the present study are available from the corresponding author upon reasonable request.
